# An In Situ Characterisation Method for 3-D Electrospun Foams

**DOI:** 10.3390/nano15050339

**Published:** 2025-02-22

**Authors:** Kyriakos Almpanidis, Chloe J. Howard, Vlad Stolojan

**Affiliations:** Advanced Technology Institute, University of Surrey, Guildford GU2 7XH, UK; k.almpanidis@surrey.ac.uk (K.A.); c.howard@surrey.ac.uk (C.J.H.)

**Keywords:** electrospinning, foam formation, foam quality, signal processing, evaluation parameters, Taguchi space design

## Abstract

Three-dimensional electrospun foams are emerging in a diversity of applications. However, their characterisation involves procedures to calculate fibre diameter and porosity, which take considerable time. Hence, in this paper, an in situ characterisation method is presented based on signal features of the grounding voltage. These features are combined into the in situ evaluation parameter **S**_r_ for each run r. The L9 Taguchi method was utilised to minimise the total number of experiments. Moreover, to prove the accuracy of this method, the traditional post-fabrication analysis was conducted, and the post-fabrication evaluation parameter was retrieved **Q**_r_ for each run r. The analysis shows that both parameters detected the same experiment run as the optimal one (with an adjusted R^2^ = 0.84) for polystyrene electrospun foams for two solution concentrations: 15%wv (run 3 with mean **S**_3_ = 54.49 and mean **Q**_3_ = 0.248) and 20%wv (mean **S**_5_ = 2.49 and **Q**_5_ = 0.248), respectively. Also, the statistical analysis shows low standard deviations for the optimal and near-optimal runs, proving the method’s repeatability. Furthermore, a theoretical explanation is provided for selecting signal features based on the Maxwellian equivalent circuit approach for the electrospun jet. Finally, this fast in situ evaluation method can replace the post-fabrication time-consuming one. It can be used as a fundamental step for an intelligent artificial intelligence tool that predicts optimal foam formation.

## 1. Introduction

Three-dimensional nanofibre foams are becoming increasingly popular in medicine [1], agriculture [2], filtration [3], and vehicle protection [4]. Their highly porous structures promote absorbance in aqueous environments, while the nanofibres’ high surface-to-volume ratio enhances reactivity with their environment [5]. Also, 3-D electrospun foam polymeric matrices were shown to achieve useful conductance, porosity, and absorbance levels. However, traditional methods of characterising these foams, such as scanning electron microscopy and complex porosity measurements, are time-consuming and only allow an assessment of the quality in the post-production stage [6]. Moreover, electrospinning is a multi-parameter-dependent production method [7], where electrostatic, environmental, and viscoelastic phenomena contribute to the final nanofibre characteristics, e.g., diameter; therefore, the serial experiment conduction is also time-consuming.

Design of a fast in situ characterisation method requires an understanding of the mechanisms (environmental, chemical, and electrostatic) that lead to foam formation. Several researchers have provided experimental explanations for foam formation with post-fabrication characterisation. Cheng et al. showed that as humidity increases, the number of reduced charges decreases; hence, the fibre diameter is increased because of the reduced elongation [8]. Moreover, Howard et al. proved the impact of the polymer concentration, humidity, temperature, and electrospinning conditions for the initiation likelihood of the 3-D electrospinning and the fibres’ diameters [9].

Sun et al. showed that negative charges generated to the foam tip attract the coming parts of the electrospun jet (positively charged jet due to positively charged needle) [10].

Cai et al., using a negatively charged needle and a positively charged collecting plate, showed that 3-D formation occurs when fibres have low surface resistivity, and charges can travel to the plate. Thus, positive charges travel to the fibre and attract the new parts of the jet while repulsing from the plate simultaneously; hence, 3-D formation was achieved [11]. Yousefzadeh et al. claimed that the first layers of the deposited foam can discharge rapidly, but the rest of the layers discharge slower because of the low conductivity polymer fibres [12]. The new fibres are accumulated rapidly vertically, and foams are built. Their explanation of formation is similar to that of Cheng et al. [8].

Electrospinning jet charge models will be introduced since electric transport to the ground significantly affects any type of deposition. Researchers tried to quantify the jet’s charge and dynamics with microscopic [13,14] and macroscopic [15,16] equivalent models. The most common microscopic (viscoelastic) model for the jet is that it behaves as a Maxwell material. This Lagrange model splits the jet into charged beads. Each bead’s acceleration depends on the field force, the viscoelastic force, the surface tension, and the coulombic forces with its neighbouring beads. The equivalent circuit for this interaction between the beads is an R-C in-series circuit. In other words, when a strain (elasticity, similar to capacitor charging) change occurs, a part of that turns into strain (due to friction and viscosity, similar to resistance energy dissipation) [17]. The most common macroscopic (kinetics) model is related to the two parts of the jet: the resistive and the convective flow. Initially, near the Taylor cone, the jet is accelerated, and the trajectory is a straight line; as it moves further from the needle, the electric field decreases, and thus the jet decelerates. When field forces are compensated by the viscoelastic and surface tension forces, jet deformation and solidification start; hence, charges are localised on the surface. Thus, the resistive flow is simulated as resistance (charge transmission) and the convective flow as capacitance (charges moving because of the electric field only).

**Figure 1:**
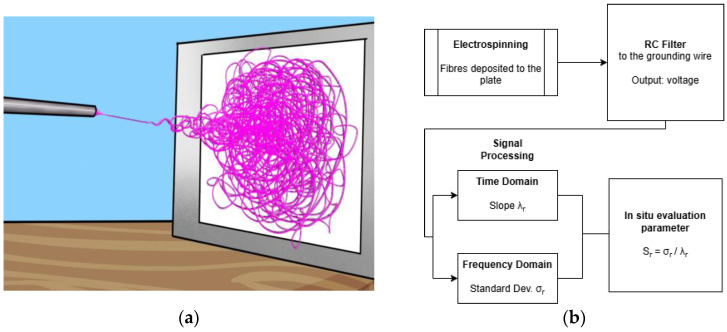
(**a**) Foam formation during 3-D electrospinning. The fibres solidified and built up in the direction normal to the collector plate. (**b**) The flow chart of the signal processing procedure for the 3D foam evaluation presented in this paper.

However, these models are related to planar (2-D) depositions only. Also, published machine learning frameworks are related to planar depositions only [18,19,20]. However, an in situ evaluation method for foam formation is still lacking in the electrospinning literature, where a global predictive method is still an open question [21]. Hence, this paper presents an in situ characterisation method for 3-D polystyrene foams (see Figure 1a) based on measurements from the grounding voltage (see Figure 1b). The grounding voltage is measured using an R-C in-parallel low-pass filter similar to the one used in [22] for 2-D depositions. The analysis is separated into two parts: (a) the grounding voltage signal processing and (b) the post-fabrication analysis based on documented experimental parameters that increase the likelihood of a foam. For both parts, an evaluation parameter is introduced. The goal is to prove the accuracy of the in situ method by detecting the same run as the optimal one with the post-fabrication method. Then, the latter method can be replaced by the former.

## 2. Materials

Polystyrene (average molecular weight (MW) 280,000 measured by the manufacturer with Gel Permation Chromatography—GPC) was purchased from Sigma-Aldrich (Gillingham, UK). Solvent Red 24 and Solvent Blue 36 (FastColours, Huddersfield, UK), N,N-Dimethylformamide (DMF), anhydrous, amine free, 99.9% (Thermo Fisher Scientific, Altricham, UK) were also utilised.

## 3. Methods

The proof of the proposed method is made by comparing the results from the in situ with the post-fabrication analysis. This comparison is achieved by introducing evaluation parameters for each analysis. In situ analysis involves monitoring the grounding voltage and retrieving signal features (signal’s slope and standard deviation) from the time and frequency domain, respectively. These features are combined into an in situ evaluation parameter **S_r_**∈R. Post-fabrication analysis involves measurements of four parameters: rate of formation, area of deposition, fibre diameter, and porosity. These parameters were normalised and combined into the post-fabrication evaluation parameter **Q**_r_ ∈R. The subscript r denotes each experiment run. Finally, the L9 Taguchi design method was used to provide the minimum feasible number of experiments, choosing the electrospinning parameters triplets (operating voltage, tip-to-collector distance, flow rate) in a way that these runs cover the whole parameter space without changing the electrospinning parameters serially.

The rest of this section is divided into subsections that describe the in situ and post-fabrication foam characterisation method and the experimental design based on the Taguchi method [23].

### 3.1. In Situ Foam Characterisation Method: Signal Processing and Parameter Derivation

The grounding signal measurements were made using an R-C in-parallel low-pass filter (nominal values: R = 1 MΩ and C = 100 nF) with a nominal cut-off frequency of 10 Hz. This filter interferes with the grounding wire, and the filter voltage V(t) ∈ R across the resistor is measured. The total signal recording was kept at 3–3.5 min for all the experiments. Voltage signal processing was conducted using MATLAB version 2023b.

#### 3.1.1. Motivation for Feature Selection

**Figure 2:**
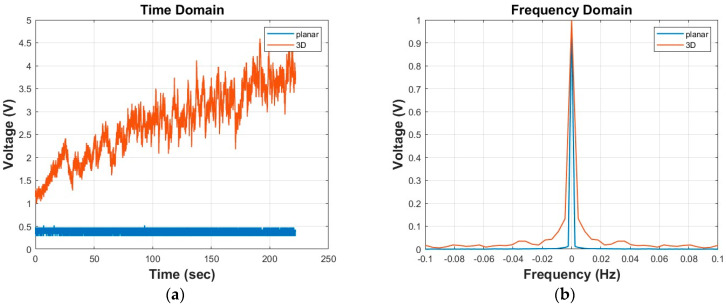
Different behaviours of the grounding signal in the 3-D formation vs. planar deposition: (**a**) the 3-D run has increasing voltage while the planar run’s voltage remains constant (time domain), and (**b**) there is a bigger accumulation of the values around zero for the 3-D run (frequency domain).

A planar and 3-D deposition with the 15%wv polystyrene solution was made. The electrospinning conditions were (12 kV, 12 cm, and 1.5 mL/h) and (15 kV, 17 cm, and 1.5 mL/h), respectively. As can be seen from Figure 2a, the grounding voltage for the planar deposition is almost stable, around 0.5 V, while for the 3-D deposition run, the grounding voltage increases from 1.5 V to 3V. Both voltage signals have the same duration.

Moreover, using Fourier transform, both signals were transformed to the frequency domain (V(ω) ∈ R). As can be seen from Figure 2b, the planar deposition run has a smaller value accumulation around the y-axis than the 3-D deposition run. For these observations to be valid, the spectra were normalised in the x- and y-axis. Hence, the next step is to quantify these observations and investigate if 3-D formations can be separated and if the same electrospinning run can be detected with the post-fabrication analysis as the optimal one.

#### 3.1.2. In Situ Evaluation Parameter **S**_r_ Based on Grounding Voltage Features

In this section, a quantification of the signal features mentioned in the previous section will be made. In this direction, the voltage increase in the time domain will be modelled with the first-order approximation. Hence, this increase is assumed to be linear and is quantified by the slope λ_r_ ∈ R for each experiment’s run r. Moreover, in the frequency domain, the symmetric around the y-axis hyperbolic Fourier transform profile is beneficial to quantify the accumulation around the y-axis with the standard deviation σ_r_ ∈ R for each experiment run r.

Considering the motivation above, the foam formation tendency derivation is based on the grounding voltage features as the greater slope (greater increase) in the time domain and the smaller standard deviation in the frequency domain (greater accumulation). Hence, the in situ evaluation **S**_r_ ∈ R parameter is defined as follows:

**S**_r_ = σ_r_/λ_r_ (1)

The in situ evaluation parameter’s optimal tendency is that the smaller, the better to avoid any numerical noise from relatively big slope values. The division function was preferred instead of, i.e., the sum of squares to penalise runs where foam formation does not happen (near-zero slope = infinite evaluation factor).

### 3.2. Post-Fabrication Foam Characterisation Method: Feature Selection and Parameter Derivation

In this section, the post-fabrication features and their effect on the electrospinning and foam formation process will be presented. These include the rate of foam formation, area of deposition, fibre diameter, and porosity. Finally, the post-fabrication evaluation parameter is introduced.

#### 3.2.1. Rate of Foam Deposition

Direct electrospinning of 3-D foams is differentiated only from traditional electrospinning by the significant formation in the third dimension [8,9,10,11,12]. The higher the rate at which the build-up of material occurs, the better the parameters for foam formation are and allow for its quantification. To measure this rate, the electrospinning process was filmed from the side, and the cross-sectional area of the foam was captured from each frame at 30-s intervals using ImageJ software (version 1.54g). By using the cross-sectional area, it gives a better representation of all material deposited. The rate of formation was then calculated using Origin 2020 software.

#### 3.2.2. Area of Foam Deposition

The area of deposition was chosen to cover all dimensions of formation. A smaller area of deposition means a faster build-up of polymer toward the needle tip, improving the rate of formation in the third dimension due to the same volume of material depositing in a smaller area. A planned photograph of the foam was taken and calculated using ImageJ software to measure the area.

#### 3.2.3. Foam Fibre Diameter

Higher concentrations of electrospun solutions generally lead to increased fibre diameter within the resulting foam [24]. The increased fibre diameters lead to enhanced mechanical support. Thicker fibres offer better structural integrity, enabling them to self-support during the 3D formation process within the foam. Furthermore, previous research has demonstrated a positive correlation between fibre diameter and 3D structure formation in electrospun materials [9]. However, this reduces the surface area-to-volume ratio. Therefore, fibre diameter was chosen as a useful parameter for assessing foam quality, with larger diameters potentially indicating successful foam formation. Fibre diameters were measured using a field-emission Scanning Electron Microscope (SEM), specifically a Tescan Mira II (Tescan, Brno, Czech Republic). ImageJ software was then used to measure the fibre diameters. Finally, Matlab2023b software was used to obtain the mean fibre diameters from the analysed data.

#### 3.2.4. Foam Porosity

Porosity is a crucial indicator of foam quality. Increased porosity leads to lower density and enhanced absorption capabilities, improving functionality in applications like filtration and cell culture [25,26,27,28].

**Figure 3:**
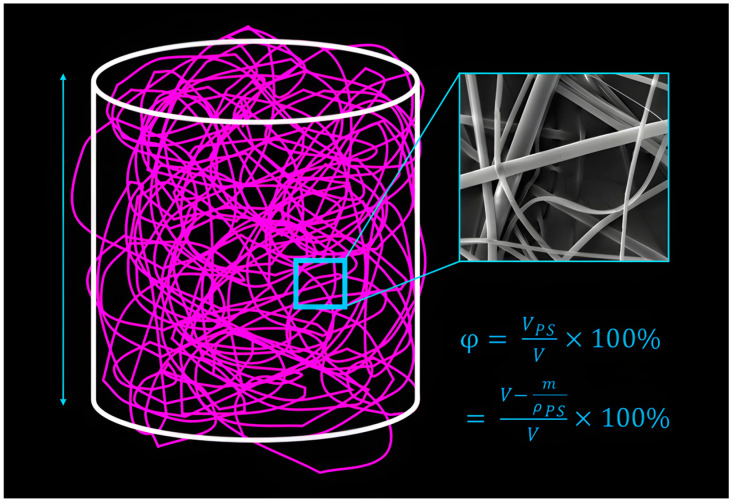
Foam characterisation of porosity and fibre diameter.

To determine the porosity ϕ ∈ R of the foam, a cylindrical geometry was adopted for ease of calculation (see Figure 3). The foam was submerged in water and gently fitted into a 5 mL syringe sleeve, approximating a cylindrical volume (V) with minimal compression. Compression will reduce the sponge porosity, leading to underestimated results. To preserve foam dimensions while removing water content, the submerged foam was frozen and then freeze-dried using a Lablyo Mini freeze dryer (Frozen in time, York, UK) for 48 h. The dehydrated foam’s outer dimensions were measured with a micrometre, achieving an accuracy of approximately 5. Subsequently, the dry foam mass (m) was measured and compared to the mass of the same volume of bulk polystyrene (ρ_bulk_·V). Finally, the porosity was calculated using the equation: ϕ = (1 − m/(ρ_bulk_·V))·100%.

#### 3.2.5. Post-Fabrication Evaluation Parameter **Q**_r_

The quality factor **Q**_r_ ∈ R of each run r was calculated using the equation below.

(2) $$Q_{r}=(A_{r}^{-1}/A_{r,max}^{-1})\cdot (R_{r}/R_{r,max})\cdot (d_{r}/d_{r,max})\cdot (\phi_{r}/\phi_{r,max}),$$

where A_r_ is the deposition area, R_r_ is the deposition rate, d_r_ is the mean fibre diameter, and f_r_ is the mean porosity of each run r, respectively. It can be observed that the deposition area feature is inversed. A smaller deposition area leads to a higher rate of deposition, which means localisation of the coming jet. Therefore, for this parameter, a lower value was considered more favourable. Before calculation, a challenge arose due to the differing units of each measured parameter. All parameter values were normalised by dividing them by the highest observed value within the whole data set to address this. This process ensures all parameters are on like scales, enabling meaningful comparisons and analysis. The post-fabrication evaluation parameter’s optimal tendency is that the greater, the better.

### 3.3. L9 Taguchi Design for Electrospinning Parameters, Solution Preparation, and Setup

Polystyrene was chosen as the polymer due to its previous success in forming 3D electrospun foams, or electrospun structures with increased thickness [10,29,30,31]. Polystyrene has demonstrated one of the highest formation rates, which will allow for better differentiation between results [9,10,11,12]. Additionally, polystyrene is a widely available polymer with numerous examples of electrospinning [30,32]. Its foams have many uses, such as oil collection from oil spill removal [33,34] and in situ cell culture [30,35,36].

Hence, polystyrene was dissolved in N,N-Dimethylformamide (DMF) to produce two concentration solutions: 15%wv and 20%wv, respectively. For each concentration solution, the electrospinning parameters have three values, as shown in Table 1 below. The parameters for each solution were chosen by finding one triplet of electrospinning parameters that resulted in foam formation and setting up the Taguchi analysis based on this case [23]. Hence, only 9 experiments will be conducted for each case study instead of 27. The parameters for each run for both solutions and also more information about the Taguchi method are given in Appendix A. Finally, each run is repeated three times to prove the reproducibility of the in situ method.

The higher concentration PS solution was made at 15 and 20%wv in N,N-Dimethylformamide (DMF) with constant stirring for 4 h at room temperature. The higher molecular weight and concentrations increase the fibre diameter and improve the mechanical strength of the fibres [24]. The 15%wv solution had 0.5%wv of Solvent Red 24 added to it, and the 20%wv solution had a 50:50 ratio of Solvent Red 24 and Blue 36 added at the same concentration used to improve the contrast of the foams for analysis.

This addition allowed imaging software to better distinguish the foam that formed. The solution was placed in a 20 mL syringe and fitted into a syringe pump. The solution was pumped through a stainless steel 21-gauge needle, and the flow rate was controlled using the WinPumpTerm v0.6 beta (Word Precision Instruments Ltd., Hertfordshire, UK). A high potential field was generated using a high-voltage DC power supply (Glassman FJ60, Gloucester, UK). The collector consisted of flat surface stainless steel covered with silicone release paper. The 15%wv solution was electrospun at ambient room temperature. For the 20%wv solution, the humidity was kept between 55 and 65% RH by boiling water inside the electrospinning chamber. The humidity and room temperature were monitored using an RS hygrometer (RS Components, Northants, UK) inside the chamber.

## 4. Results

This section will present results from the two Taguchi case studies. This section is separated into the results from the in situ method based on the **S**_r_ from Equation (1), the post-fabrication method based on the **Q**_r_ from Equation (2), and finally, a comparative analysis of these two analyses to prove that they detect the same run as the optimal one.

### 4.1. In Situ Evaluation Methodinclu

**Figure 4:**
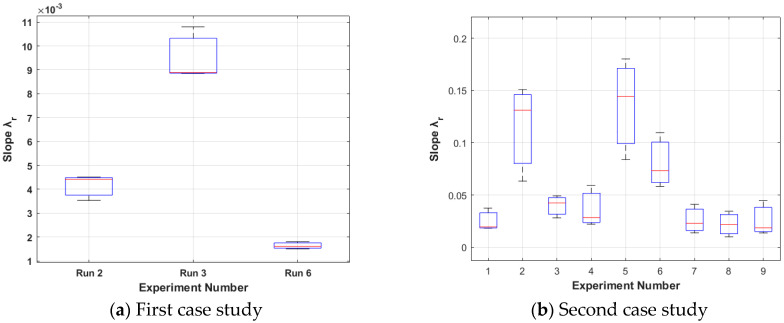
Standard deviation λ_r_ for each case study. The less the optimisation tendency is, the better. (**a**) Run 3 (12 cm, 17 kV, 4.5 mL/h) is optimal with a mean of 0.485; (**b**) run 5 (13 cm, 15 kV, 4.3 mL/h) is optimal with a mean of 0.3895. The second study resulted in greater slopes due to increased humidity, which led to a faster antisolvent effect. The red line is the mean value of each boxplot.

**Figure 5:**
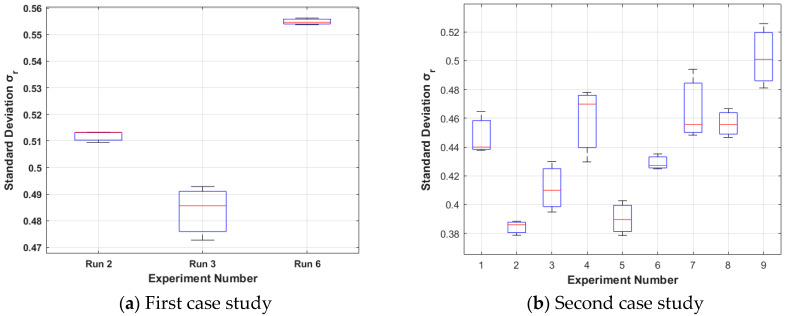
Standard deviation σ_r_ for each case study. The less the optimisation tendency is, the better. (**a**) Run 3 (12 cm, 17 kV, 4.5 mL/h) is optimal with a mean of 0.485; (**b**) run 5 (13 cm, 15 kV, 4.3 mL/h) is optimal with a mean of 0.3895. The red line is the mean value of each boxplot.

**Figure 6:**
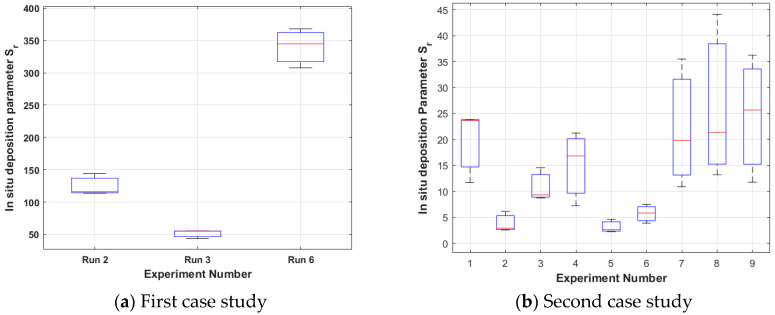
In situ evaluation parameter **S**_r_ for each case study. The less the optimisation tendency is, the better. (**a**) Run 3 (12 cm, 17 kV, 4.5 mL/h) is optimal with a mean of 54.49; (**b**) run 5 (13 cm, 15 kV, 4.3 mL/h) is optimal with a mean of 2.69. Although the boxplot overlaps, runs 3 and 5 have the smallest **S**_r_ parameters for all three repeats of each run. The red line is the mean value of each boxplot.

**Figure 7:**
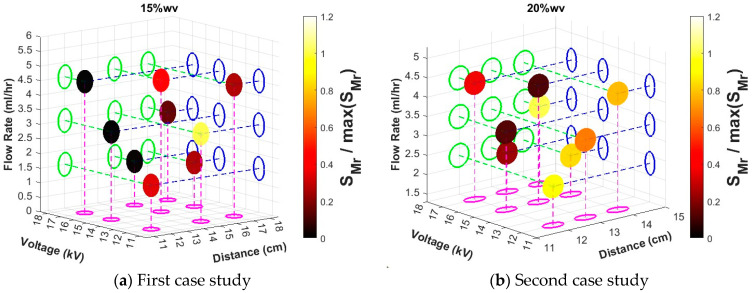
Taguchi experiment space for each set of experiments. The mean evaluation parameter of each run **S**_Mr_ was normalised and is depicted in the colour of each sphere. The optimisation rule is that the lesser, the better. Both experiments, (**a**) 15%wv solution and (**b**) 20%wv solution have their optimal case in the same region of the cube (upper left).

The results from the first case study (15%wv solution, room humidity) are presented in Figure 4a, Figure 5a, Figure 6a and Figure 7a. Boxplots are used to signify that each run is repeated three times. It can be noticed that only runs 2, 3, and 6 are presented because these runs resulted in foam formation (the others were penalised in the way described in Section 3.1.2).

Among these, run 3 is the best case (the smallest **S**_3_ for all the three repeats) with the greatest mean slope λ_M3_ equal to 0.0089 (see Figure 4a) and the smallest mean, standard deviation σ_M3_ equal to 0.485 (see Figure 5a), which leads to the smallest mean **S**_M3_ equal to 54.49 (see Figure 6a). The electrospinning parameters for run 3 are (12 cm, 17 kV, 4.5 mL/h). Moreover, as seen in the Taguchi space from Figure 7a, run 3 is on the point of the upper left edge of the cubic.

The results from the second case study (20%wv solution, high humidity) are presented in Figure 4b, Figure 5b, Figure 6b and Figure 7b. In this case study, all the runs resulted in foam formation. Among these, run 5 is the best case (the smallest **S**_5_ for all the three repeats) with the greatest mean slope λ_Μ5_ equal to 0.1444 (see Figure 4b), the smallest mean, standard deviation σ_Μ5_ equal to 0.3895 (see Figure 5b), which leads to mean **S**_Μ5_ equal to 2.69 (see Figure 6b). The electrospinning parameters for run 5 are (13 cm, 15 kV, 4.3 mL/h). Moreover, as seen in the Taguchi space from Figure 7b, run 5 is on the point of the upper middle part of the cubic.

### 4.2. Post-Fabrication Evaluation Method

The 15 and 20%wv solutions were characterised separately. Only runs 2, 3, and 6 of the 15%wv solution exhibited three-dimensional characteristics; the two-dimensional solutions only allowed for fibre diameter measurements. The 20%wv solutions all had centralised formation, and increased solution concentration and humidity allowed for formation in all conditions [9].

**Figure 8:**
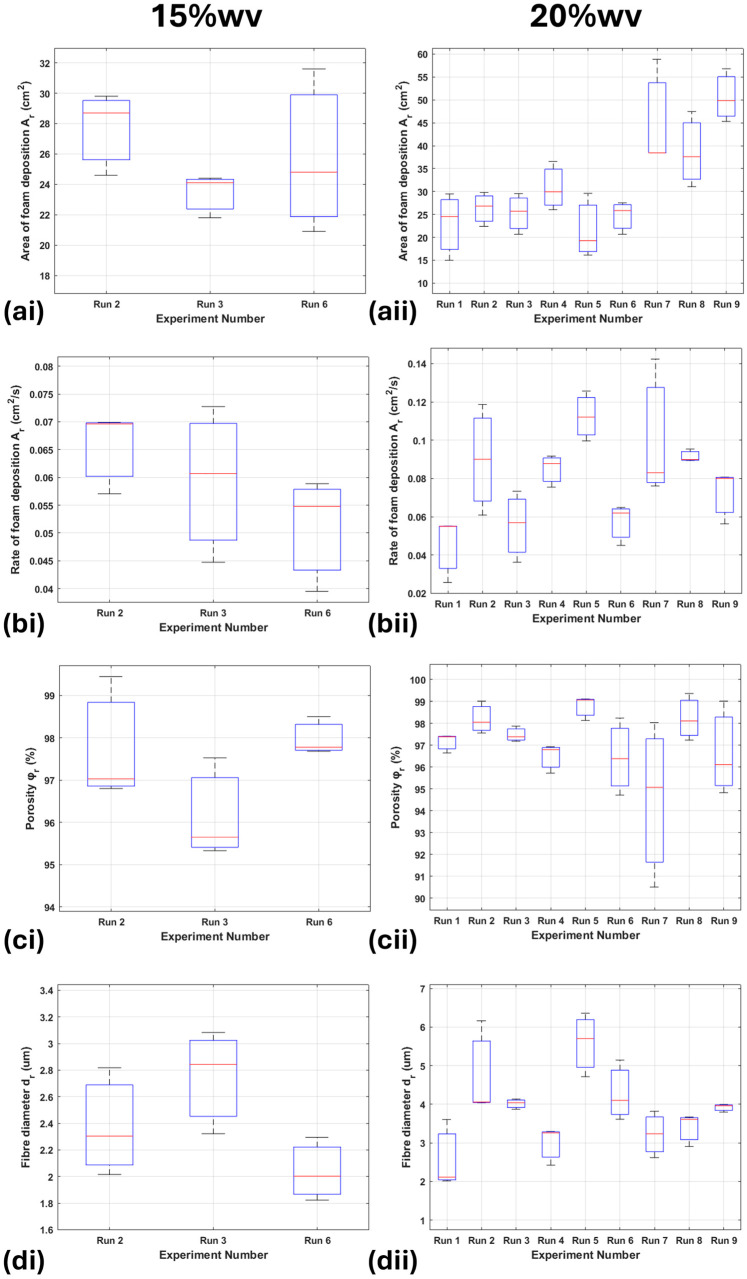
The red line is the mean value of each boxplot. In the left hand side panels, showing boxplots for the 15%wv solution for the (**ai**) foam area, (**bi**) foam deposition rate, (**ci**) foam porosity and (**di**) fibre diameter we cannot detect, we cannot detect an optimum between the sets of experimental conditions that lead to foam production. This is the same for the 20%wv solution, where there may be an indication that the (**aii**) the area of deposition increases for the latter runs, however (**bii**) the rate of foam deposition, (**cii**) the porosity and (**dii**) the fibre diameter do not show a trend.

The area of deposition shows a general trend of increase with distance from the needle tip (Figure 8(ai,aii)). This trend is less prominent for the 15%wv solution. The deposition rates for the 20%wv also show a trend with increasing distance (Figure 8(bi,bii)); by calculating the percentage contribution using Minitab (Version 22.1.0), it shows a value of 77.7%; however, runs 2 and 5 show a rate above their trend level, suggesting a promising increase in foam quality. The foam’s porosity is all above 94%, with runs 2, 5 and 8 for the 20%wv solutions reaching >98% porosities close to ultralight status (Figure 8(ci,cii)). The voltage affects the porosity of the foams the most, with a percentage contribution of 73.5%. The 15%wv fibre diameters significantly increase when foam deposition occurs (Figure 8(di)).

After running a paired sample *t*-test at level 0.05, it was found that the increase in fibre diameter in foam deposition was significant (*p*-value equals 0.0054). The 20%wv solution shows a higher fibre diameter, which is not only due to the full foam deposition but also the increased solution viscosity and humidity [9,10]. Runs 2 and 5 show the highest fibre diameters (Figure 8(dii)), but the results in this figure do not show a clear tendency for the optimal case.

**Figure 9:**
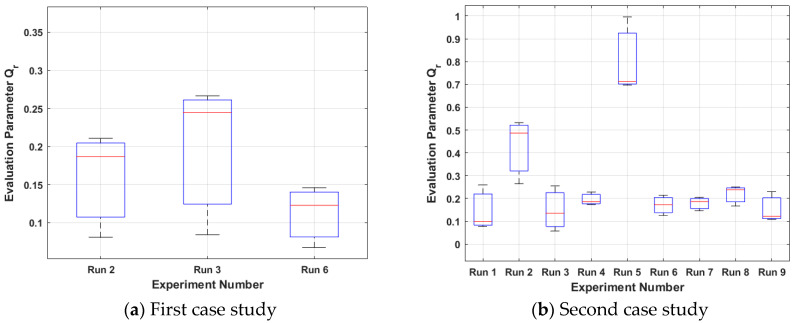
Quality factor **Q**_r_ for each set of experiments. The greater the optimisation tendency, the better. (**a**) Run 3 (12 cm, 17 kV, 4.5 mL/h) is optimal with a mean of 0.248; (**b**) Run 5 (13 cm, 15 kV, 4.3 mL/h) is optimal with a mean of 0.71. The quality factor indicates the best case for each solution because the resistance and capacitance change values when the geometrical parameters charge; hence, the full set of parameters must be considered. The red line is the mean value of each boxplot.

The characterisation results were normalised, and the mean quality factors (**Q**_r_) were calculated using Equation (1). Runs that did not produce foam for the 15%wv solution have a quality factor of 0 due to certain parameters not being quantified (e.g., the rate of formation cannot be measured for two-dimensional sheets). The optimal case for 15%wv is run 3 (12 kV, 15 cm, 3 mL/h), as shown in Figure 6a. In general, the quality factors for 15%wv are lower than 20%wv, which can be attributed to the higher solution concentration and humidity. Run 5, followed by run 2, shows a significantly higher quality factor (Figure 9b), which, excluding the previously mentioned factors, seems to be due to the high contribution of the voltage at 57.0%.

### 4.3. A Comparative Analysis Between the In Situ and the Post-Fabrication Methods

**Figure 10:**
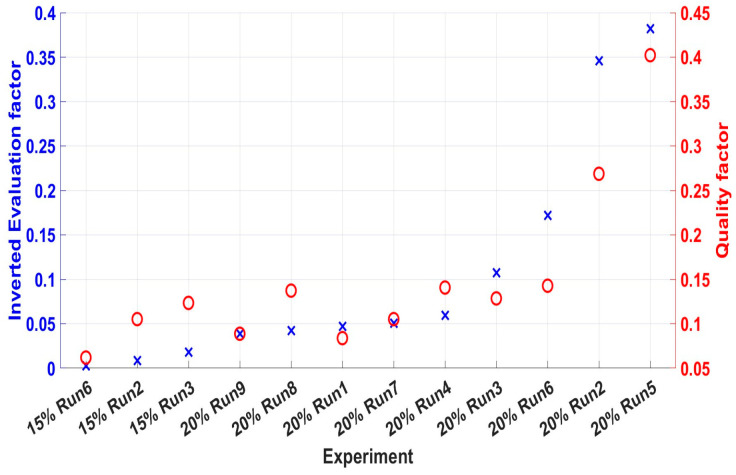
The mean inverted evaluation parameter 1/**S**_Mr_ (crosses) and the mean quality factor **Q**_r_ (open circles) for each run. For the first case study, these two parameters follow the same tendency. For the second case study, only the first three ones follow the same tendency. The in situ method can predict the optimal run for both case studies.

In this subsection, both analyses (in situ and post-fabrication) for the two case studies will be compared. Since optimal and near-optimal runs have low standard deviations, the mean value **S**_Mr_ and **Q**_Mr_ of each run r will be utilised for a comparative study. Figure 10 presents the inverted values of each mean **S**_Mr_ and **Q**_Mr_. Each run’s ID is inserted into the horizontal axis so the SMr can be in ascending order. After that, the **Q**_Mr_ was inserted for each run to detect the correlation between **S**_Mr_ and **Q**_Mr_. The mean in situ evaluation parameter **S**_Mr_ is inverted to have the same optimisation tendency as **Q**_Mr_ (the greater, the better).

As can be seen from Figure 10, both analyses detected run 3 and 5 as the optimal cases for the two case studies, respectively. Also, run 5 of the second case study is the best formation among all the runs. Moreover, for the first case study, **S**_Mr_ and **Q**_Mr_ have the same tendency for all the foam formation cases. The mean evaluation parameters for the second case study follow the same tendency for the first three best cases. Lastly, the two datasets are correlated with adjusted R^2^ = 0.84.

## 5. Discussion

In this section, a discussion of the in situ and the post-fabrication methods will be provided. Physical meaning, explanation, and comments on the methods’ stabilities and robustness will be provided.

### 5.1. In Situ Evaluation Method: **S**_r_ Physical Meaning, Formation Explanation, Method’s Stability

The in situ evaluation method can effectively predict the same case as optimal with the post-fabrication method. The slope for the second case study was much higher than the ones calculated for the first case study (see Figure 4). This can be attributed to the higher humidity, resulting in a higher antisolvent effect, fibre solidification, and, thus, 3-D foam formation. Furthermore, the optimal runs for each case study (runs 3 and 5) have smaller evaluation parameters for each run repeat, although the phenomenal boxplot overlaps. Moreover, for the first case study, each slope and standard deviation have the same tendency as the evaluation factor. There are deviations among the boxplots and repeats for the second case study. This can be attributed, again, to the higher humidity and the increased stochasticity of charge transport to the ground [37].

The **S**_r_ is an evaluation parameter and has a physical meaning. Slope λ_r_ is related to the foam formation, which is opposite to the direction of the positively charged needle. As the jet parts continuously come, this means that the foam tip crosses the electric field’s equipotential lines. Hence, the slope of the measuring voltage increases.

For the standard deviation σ_r_ physical meaning, the foam formation is assumed to be represented as an R-C in-series equivalent circuit. This can be explained as the foam tip having negative charges either because they screen the positive field or are reoriented inside the fibre, as stated in [38,39]. These negative charges attract the coming jets to the foam tip. Moreover, the needle’s positive charges and the foam tip’s negative charges create capacitance. On the other hand, the foam body has positive charges that travel to the grounding place, initially through the foam matrix. However, the low conductivity of the fibres creates repulsive forces among the fibres, creating a resistance increase. Hence, this capacitance and resistance increase can be correlated with a decrease also in the cut-off frequency of the R-C foam formation equivalent circuit.

Moreover, the foam formation is termed as the regime where the height of the deposited nanofibres increases at rates of the order of cm/min, rather than mm/h (because of the charges’ regime mentioned above [38,39]—negative charges on the tip, positive to the body) and where the aspect ratio of height to base area diameter is ~1–3, as opposed to <0.01, as would be obtained for 2D films [10,11,31]. The transition regime between film and foam is not necessarily sharp or well defined, and further research may be needed to accurately define and describe this regime.

Also, it was observed that sometimes, during foam formation, discharge phenomena occurred. These leads to a short-term collapse of the sponge. These phenomena can be attributed to the positive charge travel to the ground either via air/humidity ionisation [37] or by exceeding the foam body’s dielectric strength [31].

Lastly, the R-C foam formation circuit is assumed to be the dominant to any other phenomena that may occur during electrospinning but also dominates the R-C in the parallel filter. Theoretically, the filter’s time constant is 0.1 s, and the foam formation started more than 10–15 s after electrospinning started. This means the capacitance was already saturated, and the near-DC signal comes from the current that passes through the resistance. This is verified by the fact that the output signal is in the order of several volts (1–20 V) and the input signal is more than 12 kV.

### 5.2. Post-Fabrication Evaluation Method: Robustness and Experimental Observations

**Figure A1:**
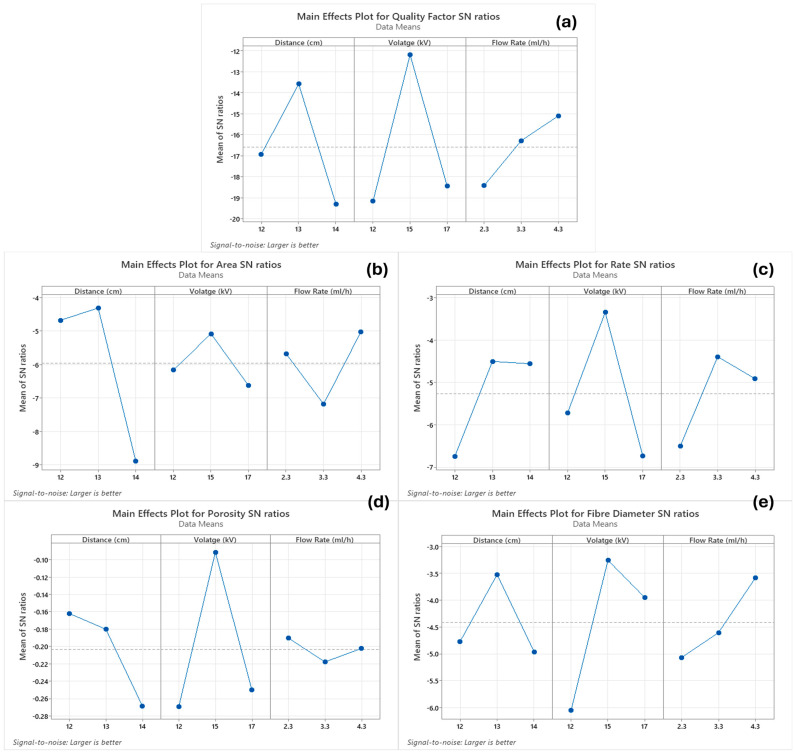
The main effects plot for the 20%wv solution. The peak of each plot shows the optimal conditions for each parameter. The dashed lines are the mean SN ratios. (**a**) Voltage is the dominant factor impacting the quality factor). The optimal condition, matching the signal analysis result, is the triplet (13 cm, 15 kV, and 4.3 mL/h). (**b**) The distance has the greatest influence on the area of deposition. Voltage is the dominant influence parameter for the deposition rate (**c**), porosity (**d**), and fibre diameter (**e**).

The quality factor effectively combines characterisation techniques, demonstrating its usefulness in identifying well-supported sponges with porosities greater than 95% and fast production speeds. While these factors are crucial for many applications, some may necessitate additional or different parameters, such as cell attachment [40], as an additional quality measure. Further research is needed to tailor this technique to specific applications. Ignoring the influence of concentration and humidity as these acted as controls, voltage emerges as the dominant factor impacting the quality factor (Figure A1a). The optimal condition, matching the signal analysis result, is the triplet (13 cm, 15 kV, and 4.3 mL/h). This dominance of voltage is further evident in the wider value ranges observed for the deposition rate (Figure A1c), porosity (Figure A1d), and fibre diameter (Figure A1e) across the voltage range. The area of deposition exhibits a distinct trend, with distance having the greatest influence (Figure A1b), an observation previously observed in [41].

The potential sources of error in the quality factor include foam compression during porosity measurement, leading to underestimated porosities. Additionally, measuring deposition in only one plane could introduce errors; ideally, the deposition should be measured in all dimensions. However, these systematic errors are present across all foam runs, and normalisation should minimise their impact on the final score. The final introduction of error may be due to the non-stabilised humidity measurement due to equipment limitations. The run repeats help negate errors by treating changes as background noise.

Additionally, the 20%wv set of experiments all produced foam compared to the 15%wv due to the high solution concentration related to higher viscoelasticity, higher surface tension, the pairing of the macromolecular chains, and the stretching of the jet [9,24]. The high humidity results in an antisolvent effect because of the increased interaction with the water’s molecules [10]. This interaction diminishes the elongation of the electrospinning jet, resulting in larger fibre diameters. These parameters were shown to improve sponge quality substantially. The relation of the post-production foam characteristics to the signal processing features can be found in Table 2.

## 6. Conclusions

In this paper, an in situ evaluation method for 3-D electrospun foams was presented. The filtered grounding voltage can be used as an alternative to the time-consuming post-fabrication analysis. Both methods have detected the same case as optimal: (a) run 3, for the 15%wv solution (mean **S**_3_ = 54.49 and mean **Q**_3_ = 0.248) and (b) run 5 for the 20%wv solution (mean **S**_5_ = 2.49 and **Q**_5_ = 0.248). The in situ and post-fabrication evaluation methods are correlated with an adjusted R^2^ = 0.84. The reason for this is based on the fact that the signal processing features are related to electrostatic and electric transport phenomena that happen during the foam formation. This evaluation method can be a candidate as a part of an intelligent software decision-making algorithm for finding the optimal foam formation electrospinning conditions. Future work will involve the testing and evaluation of the proposed in situ evaluation method in various polymers to build a neural network for further signal feature detection and extraction. The proposed method can broaden the 3-D electrospinning state-of-the-art knowledge for artificial intelligence (AI) specialists and signal processing engineers; hence, they can contribute with their ideas to build an accurate, intelligent electrospinning model that predicts the parameters for optimal formation.

## Figures and Tables

**Table 1:** The parameters used for the Taguchi design and their values for each set of experiments. Only three parameters were chosen to simplify the analysis. These are the simplest to alter between experiments.

Case Study	Voltage (kV)	Tip-to-Collector Distance (cm)	Flow Rate (mL/h)
15%wv Room humidity	12	12	1.5
	15	15	3
	17	17	4.5
20%wv High humidity	12	12	2.3
	13	15	3.3
	14	17	4.3

**Table 2:** Foam structural characteristics and their connection to grounding signal.

Characteristic	Role	Connection
Rate of formation	The stronger attraction of the jet to the already-formed sponge.	The virtual capacitance changes faster, and the equipotential lines’ rate of crossing—a greater slope and cut-off frequency.
Area of formation	Centralised formation leads to centralised negative charges.	Increases the current spatial density and the crossing of the equipotential lines—greater slope.
Fibre diameter	Thicker nanofibres promote integrity and 3-D formation.	Reduced resistivity for the charges going to the ground—greater slope.
Porosity	Increased porosity promotes absorption and cell culture.	Connected nanofibres—both greater slope and cut-off frequency.

## Data Availability

These data are available upon request.

## Appendix A

The Taguchi method is traditionally used to optimise products and processes, ensuring consistent performance even in the presence of uncontrollable factors. In electrospinning, the environment (e.g., humidity and temperature) is the primary source of such factors [23]. To achieve this, an orthogonal array is designed, allowing for the efficient exploration of many factors with a minimal number of experiments. This array facilitates the identification of optimal conditions from the resulting data set [42]. While traditional characterisation methods can be time-consuming, the Taguchi method significantly reduces the time spent while still identifying optimal conditions for comparison [43,44].

In this direction, the L9 Taguchi orthogonal array is a Design of Experiment approach where three parameters, with three levels each are investigated with nine experimental runs, rather than the 3 × 3 × 3 = 27 full set. This is because to project the 3 × 3 × 3 data set onto each pair of parameter axes, only 9 unique points in the 3D data set are needed, with the other 18 being redundant. For each solution concentration, an L9 Taguchi orthogonal array was created (Table A1 and Table A2). Three factors—voltage (kV), tip-to-collector distance (cm), and flow rate (mL/h)—were compared at three levels.

**Table A1:** Case study 1: Taguchi orthogonal array.

Run	Operating Voltage (kV)	Tip-to-Collector Distance (cm)	Flow Rate (mL/h)
Run 1	12	12	1.5
Run 2	12	15	3
Run 3	12	17	4.5
Run 4	15	12	3
Run 5	15	15	4.5
Run 6	15	17	1.5
Run 7	17	12	4.5
Run 8	17	15	1.5
Run 9	17	17	3

**Table A2:** Case study 2: Taguchi orthogonal array.

Run	Operating Voltage (kV)	Tip-to-Collector Distance (cm)	Flow Rate (mL/h)
Run 1	12	12	2.3
Run 2	12	15	3.3
Run 3	12	17	4.3
Run 4	13	12	3.3
Run 5	13	15	4.3
Run 6	13	17	2.3
Run 7	14	12	4.3
Run 8	14	15	2.3
Run 9	14	17	3.3
